# Hospital Expenditure. — The Commissariat

**Published:** 1895-11-09

**Authors:** 


### The Institutional Workshop.

### HOSPITAL EXPENDITURE.—THE COMMISSARIAT.

### V.—THE PROPORTION OF SERVANTS.

Every household has its own way with regard to the staff of servants, and in no point is variation of hospital administration more clearly shown than in this particular. The servants differ in respect to their numbers, duties, and description; in the amount of board they receive, the length of time they remain on duty, and in sleeping in or outside the premises. Needless to say, also, they vary widely in the amount of wages received. The following table gives the proportion of the patients to those servants who receive full board, those receiving partial board reckoning as half. The proportion is worked out, as in the case of the nurses, on the basis of the occupied beds, fractions omitted:—

| London. | Average of occupied beds to each servant boarded. |
|---|---|
| University | 26 |
| *Westminster | 14 |
| Guy's | 12 |
| London | 9 |
| St. Thomas's (not including Nightingale Home) | 9 |
| Seamen's | 9 |
| Royal Free | 8 |
| St. George's | 7 |
| St. Bartholomew's | 7 |
| St. Mary's | 7 |
| †Middlesex | 6 |
| Charing Cross | 6 |
| German | 6 |
| North-West London | 3 |
| Great Northern Central | 3 |
| Metropolitan | 3 |
| **Provincial, Scotch, and Irish.** | |
| Birmingham, Queen's | 9 |
| Bradford | 9 |
| Dublin, Meath | 9 |
| Hull Royal Infirmary | 6 |
| Newcastle Royal Infirmary | 6 |
| York County | 6 |
| Gloucester General | 6 |
| Edinburgh Royal Infirmary | 6 |
| Glasgow Royal Infirmary | 6 |
| Glasgow, West, Infirmary | 6 |
| Aberdeen | 6 |
| Birmingham General | 5 |
| Bristol General | 5 |
| Halifax | 5 |
| Leeds General | 5 |
| Norfolk and Norwich | 5 |
| Sheffield General | 5 |
| Derbyshire Royal Infirmary | 5 |
| Wolverhampton | 5 |
| Ayr County | 5 |
| Dundee | 5 |
| Belfast, Royal | 5 |
| †Cambridge, Addenbrooke | 4 |
| Liverpool Royal Infirmary | 4 |
| Oxford | 4 |
| Sussex | 4 |
| Kilmarnock | 4 |

* Our attention has been drawn to the fact that at the Westminster Hospital a certain number of nurses and probationers, from a nurse training institution, who were not boarded in the house, were employed in attending to the patients at the time our figures were taken. It thus happened that while, from a housekeeping point of view, which was the point discussed in our article, we were correct in giving the average of patients to each nurse as 4·6, from the nursing point of view the average was only 3·2.

† Note.—These hospitals having been closed during part of the year 1893 on which the calculations are based, the returns both as regards nurses, servants, and expenditure are exceptional.

One feature of economy in a large household is plainly revealed by these figures. The large London hospitals containing over 200 beds board something like half the number of servants in proportion to the smaller ones both in London and the provinces, while in those receiving less than 50 patients the proportion rises to one servant among three. University Cottage Hospital stands on an exceptional basis, as boarding none of the nurses and hence requiring far less labour. It is the exception in London hospitals to include the board of laundrymaids with that of the household, and these have not, therefore, been reckoned in any case. In the country the laundrymaids are almost invariably included; the rate of wages is lower, and it is less easy to get outside workers, and accordingly a far larger proportion of indoor labour is employed than in London.

Some of the divergences of custom which have arisen may be noted. Out of the London institutions the wardmaids are fully boarded in seven, and in one are partially boarded. Scrubbers commonly receive no board, but in four hospitals a few out of the number employed receive full board, and in one they are all partially boarded. There is a difference of usage, too, as to the amount of food which falls to their share in a casual way. In some institutions the no-board rule is rigidly enforced; in others any surplus from the dinners of the patients is distributed among them to carry away; in others, again, this practice is forbidden, but the prohibition is evaded. All the servants employed in the kitchen and for waiting and general housework are invariably boarded. In only five hospitals are laundrymaids fully boarded, but at Guy's they are separately lodged, and the amount of their cost is more conveniently reckoned off under the item "washing." It is usual to board some of the porters, either fully or partially, even when they do not sleep on the premises, owing to the inconvenience of having them out of the way at meal times. In several hospitals—notably the London, Middlesex, Metropolitan, and St. Mary's—the porters' table is a heavy item for the housekeeper, from seven to twelve men receiving board daily. In only three—viz., St. Thomas's, Guy's, and St. Bartholomew's—no board is allowed to porters.

In the provincial hospitals scrubbers are often boarded as well as wardmaids, and the laundry staff either reside altogether on the premises or receive full board. The porters are boarded with but one exception (Halifax), and it is very usual also to provide meals for the fireman or engineer.

In Scotch hospitals wardmaids and scrubbers are often classed together and receive full board, though not always lodging. It is easy to understand why the proportion of servants boarded in provincial institutions where space is less cramped, and there is greater facilities for lodging indoor servants, is higher than in London. The effect of the number boarded upon the total expenditure in the kitchen is not traceable from outside among so many other stronger elements in the household. It will, however, stand out very clearly in the mind of the careful housekeeper, and data are not wanting for ascertaining with some degree of accuracy later on the annual charges on the hospital establishment of this item.

